# Advanced robotics for automated EV battery testing using electrochemical impedance spectroscopy

**DOI:** 10.3389/frobt.2024.1493869

**Published:** 2025-01-10

**Authors:** Alireza Rastegarpanah, Cesar Alan Contreras, Mohamed Ahmeid, Mohammed Eesa Asif, Enrico Villagrossi, Rustam Stolkin

**Affiliations:** ^1^ School of Metallurgy and Materials, University of Birmingham, Birmingham, United Kingdom; ^2^ The Faraday Institution, Didcot, United Kingdom; ^3^ Faculty of Computing, Engineering and the Built Environment, Birmingham City University, Birmingham, United Kingdom; ^4^ Institute of Electrification and Sustainable Advanced Manufacturing (IESAM), Newcastle University, Newcastle upon Tyne, United Kingdom; ^5^ Institute of Intelligent Industrial Technologies and Systems for Advanced Manufacturing, National Research Council of Italy, Milan, Italy

**Keywords:** electrochemical impedance spectroscopy, EV battery, Lithium-ion battery recycling, admittance Control, robotic disassembly

## Abstract

**Introduction:**

The transition to electric vehicles (EVs) has highlighted the need for efficient diagnostic methods to assess the state of health (SoH) of lithium-ion batteries (LIBs) at the end of their life cycle. Electrochemical Impedance Spectroscopy (EIS) offers a non-invasive technique for determining battery degradation. However, automating this process in industrial settings remains a challenge.

**Methods:**

This study proposes a robotic framework for automating EIS testing using a KUKA KR20 robot arm mounted on a 5 m rail track, equipped with a force/torque sensor and a custom-designed End-of-Arm Potentiostat (EOAT). The system operates in a shared-control mode, enabling the robot to function both autonomously and semi-autonomously, with the option for human intervention to assume control as needed. An admittance controller ensures stable connections, with forces optimized for accuracy and safety. The EOAT’s mechanical strength was validated through finite element analysis.

**Results:**

Experimental validation demonstrated the effectiveness of the developed robotized framework in identifying varying levels of battery degradation. Internal resistance measurements reached up to 1.5 mΩ in the most degraded cells, correlating with significant capacity reductions. The robotic setup achieved consistent and reliable EIS testing across multiple LIB modules.

**Discussion:**

This automated robotic framework enhances battery diagnostics by improving testing accuracy, reducing human intervention, and minimizing safety risks. The proposed approach shows promise for scaling EIS testing in industrial environments, contributing to efficient EV battery reuse and recycling processes.

## 1 Introduction

The adoption of electric vehicles (EVs) has accelerated in the past few years, and this has been accompanied by technological advancements, policies, and environmental concerns. It has influenced the economy (Haddadian et al., 2015) with multiple challenges along the way, including End of Life (EoL) management and recycling (Steward et al., 2019). The EV batteries are deemed inappropriate as traction batteries once they reach 75%–80% of their initial rated capacity, provided the State of Health (SoH) is adequately assessed. The potential for reusing or re-purposing these batteries in other applications offers a promising avenue.

SoH is needed to determine the operational efficiency and overall remaining useful life (RuL) of a lithium-ion battery (LIB). SoH compares the current performance of a battery to its initial state. Depending on the method and literature, multiple factors can be used to describe this metric (Yang et al., 2021). Factors such as capacity fade, power fade, increase in internal resistance, charge/discharge cycles, temperatures, capacities, and currents can all influence the assessment of the SoH and in quantifying the RuL. Assessing the SoH is crucial for evaluating the operational performance, associated risks, and reuse potential of LIBs. Beyond their application in electric vehicles, LIBs are used in various domains, including renewable energy storage, portable electronics, and grid stabilisation systems (Zhao et al., 2021). Even after their primary lifecycle in EVs—where they endure rigorous operational conditions and degradation—these batteries retain a substantial portion of their initial capacity at the end of their service life (EoL), making them suitable for secondary applications. Consequently, ensuring an accurate SoH evaluation becomes one of the most critical factors for assessing the potential of these batteries in alternative applications. Recycling is another aspect of EoL of batteries. Batteries contain valuable and often scarce materials, including lithium, graphite, and cobalt. Efficient recycling processes enable the recovery of these valuable materials, which can be reused to produce new batteries or other systems. They undoubtedly will be part of maintaining a supply chain of LIBs (Pinegar and Smith, 2019). In this context, developing robust and scalable systems for testing the health of used batteries is becoming increasingly important. These methodologies facilitate efficient battery recycling and repurposing, contributing to the circular economy, particularly in heavy-duty environments.

Handling EV batteries, especially during testing and recycling, involves many safety and technical challenges. Although retired from EV use, these batteries still contain stored energy and can pose significant hazards if mishandled. In addition to physical risks like fires and explosions, EV batteries also present chemical dangers due to the materials they contain. Integrating automation into battery testing procedures offers several advantages: it improves safety by minimizing human interaction with hazardous batteries, reduces reliance on manual labour by enabling continuous operation, and enhances the accuracy and consistency of testing.

In our previous study (Rastegarpanah et al., 2021a), the use of EIS was demonstrated for automating battery health diagnostics. EIS measures a system’s resistance to an AC signal across various frequencies, providing insights into battery health and performance. A collaborative robot was equipped with custom-designed End-of-Arm Tooling (EOAT) and a potentiostat, successfully automating the EIS collection process on a single Nissan Leaf 24 kWh LIB module. This pilot work, and most of the current literature, faces the challenge ofstudy highlighted the feasibility of automating such tasks using a collaborative robot arm. The robotic testing framework utilized visual servoing (VS) to localize and align the EOAT with the battery terminals, and an impedance controller ensured stable contact for accurate data collection. Building on this idea, the current study scales the testing method from single modules to stacks of modules and extends automation from laboratory-scale collaborative robots (Rastegarpanah et al., 2021a) to industrial-scale heavy-duty robotic arms, albeit with some changes. VS has been replaced with pre-programmed positions and operator manual control, while the impedance control has been replaced with admittance control for the industrial setup.

**FIGURE 1:**
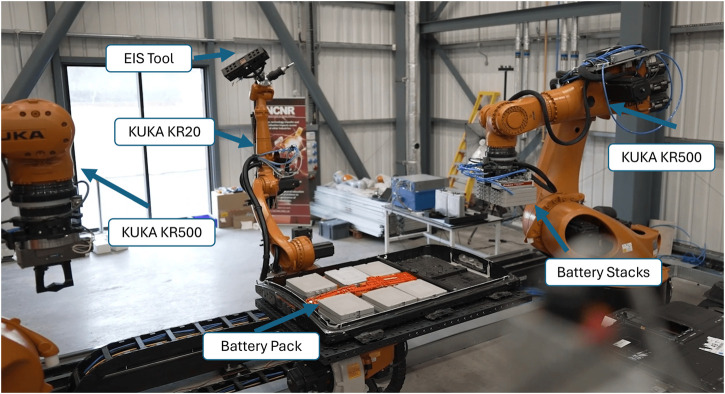
Robotic test-bed developed for testing, disassembly, and sorting of EV batteries at the Birmingham Energy Innovation Centre.

This advancement reduces the need for direct human intervention, thus minimizing health and safety risks associated with handling high-energy lithium-ion batteries. In addition, the heavy-duty arms can lift heavy objects like battery packs and stacks of modules. The EIS technique has been improved by automating the testing process using a KUKA KR20 robot arm mounted on a 5-m rail. Additionally, the Interfacing Toolbox for Robotic Arms (ITRA) (Mineo et al., 2019) was employed, which integrates industrial robotic arms with server computers, sensors, and actuators, providing easier control of the robot system, allowing a user to simply program in high-level languages and implement with libraries already developed for collaborative robot systems. The main contribution of this study includes proposing a robotic framework for EIS testing in an industrial setup, ensuring EIS testing is safer, using an admittance control when connecting the industrial arm with the battery terminals, and providing consistent results compared to traditional methods. The system’s adaptability ensures it can handle various damaged and undamaged battery conditions, addressing the increased demand generated by the growing electric vehicle industry. Figure 1 depicts our developed robotic testbed for testing, disassembly, and sorting EV batteries.

The present paper is structured as follows: Section 2 discusses related works in EV battery testing, EIS, and the use of robotics to automate such procedures. Section 3 gives a presentation of the task and the involved components, first giving a step-by-step description of the procedure, followed by presenting the hardware, tools, safety protocols, and robot control architecture used for performing the EIS data collection and testing, and finishing with an in-depth explanation of the EIS method, and the results of the tests. Section 4 presents the findings and results of the testing, analysing the obtained data and giving an analysis of it. Finally, Section 5 concludes with insights, findings, and broader implications of our research on the battery ecosystem, also talking about potential future directions in EV battery testing.

## 2 Related works

Multiple battery testing methods have been developed due to the recent necessity of reusing and disassembling EV batteries. Assessing the battery health condition is one of the primary research objectives necessary to determine the battery’s life expectancy and possible repurposing capability for second-life applications. Our previous work (Rastegarpanah et al., 2021a), and most of the current literature, faces the challenge of automating the battery testing process, where the goal is not only to assess the health of the batteries but also to do this with fully autonomous systems without putting humans in danger. Notable challenges include establishing a stable connection to the terminal to the modules of the spent LIB; given that the procedure is primarily done manually via human operator, this raises safety concerns regarding battery-related incidents that can be injurious to humans, such as electrical shocks, chemical burns, or explosions (Chen et al., 2021; Lai et al., 2022).

Several non-destructive techniques for SoH estimation in LIBs have been developed and tested in various studies. Commonly used methods include capacity-based techniques (Yang et al., 2017; He et al., 2020; Lin et al., 2022), Kalman filtering (Plett, 2004; Hossain et al., 2022; Xu et al., 2012; Li et al., 2020), EIS (Li et al., 2022; Zhang et al., 2020; Jiang et al., 2022; Middlemiss et al., 2020), and thermal analysis (Fleckenstein et al., 2013; Wang et al., 2021; Razi et al., 2021). Among these, capacity-based methods are extremely accurate but require extensive charge-discharge cycling, making them time-consuming (Jiang and Pang, 2022). In contrast, EIS offers a faster alternative by analysing the battery’s internal resistance and measuring the resulting current to construct an impedance spectrum over a range of frequencies. This spectrum reveals detailed insights into the battery’s internal processes and conditions. The data obtained from EIS can be further enhanced using neural networks or other deep learning algorithms to predict a battery’s RuL (Gao et al., 2021; Ghosh et al., 2022; Lu et al., 2022; Rastegarpanah et al., 2023; Zhang et al., 2023; He et al., 2023; Hu and Wu, 2024; Huang et al., 2024; Chen et al., 2024).

### 2.1 Battery state of health

Advancements in capacity-based methods and cycling tests for battery diagnostics and SoH analysis are widely researched, constantly improved, and preferred for battery testing despite requiring multiple charging and discharging cycles. He et al. (2020) propose a function-based voltage-capacity model that uses features of interest from their collected data and performs linear correlations to predict battery SoH. However, this method requires at least 100 cycles to make accurate predictions. Similarly, Lin et al. (2022) employ cycling and ageing experiments to develop a back-propagation neural network using current and incremental capacity data. This approach requires between 40 and 200 cycles to achieve predictions with a mean absolute error (MAE) of less than 2%. While effective, these methods are time-consuming due to the necessary cycling. The time required for N cycles depends on factors such as the charge/discharge rates (C-rates) and cycling protocol. For instance, fast cycling at 1C can take 1–2 h per cycle, whereas slower cycling at 0.5C may take 3–4 h per cycle. Long-term testing at lower C-rates (e.g., 0.1C) could extend the time to a day or more per cycle, meaning N cycles could span anywhere from days to months.

Temperature-based methods are another type of offline testing method, which requires data on ageing batteries across cycles to analyse their SoH. Gaussian Process Regressions (GPR) and differential thermal voltammetry (DTV) curves together in models by Wang et al. (2021) show it is possible to obtain an MAE of less than 2% with these models. Still, battery cycle limitations continue to exist, as in capacity-based models.

Kalman filtering techniques surpass capacity-based models and temperature-based methods in speed because they enable real-time estimation while a battery is in use, providing a significant advantage. However, they necessitate an accurate mathematical model, sequential measurements, and precise parameter estimations (Hossain et al., 2022). Enhancements to Kalman filtering, such as the Multi-innovation Extended Kalman Filter (MI-EKF) by Li et al. (2020), aim to address noise and other errors in parameter estimation. Furthermore, the introduction of the Adaptive Iterative Extended Kalman Filter has refined state of charge (SoC) estimation by dynamically updating and summing voltage iterations upon reaching certain thresholds, achieving a MAE of under 1% (He et al., 2021). Additionally, the Fractional-Order Adaptive Square-Root Cubature Kalman Filter (FO-ASRCKF) by Chen et al. has shown promise by adjusting to experimental data and noise conditions, even with incorrect initial values, improving SoC prediction accuracy and reducing the MAE to less than 0.5% (Chen et al., 2023).

Finally, EIS has been developed as a non-invasive, *in-situ* method for assessing the SoH of lithium-ion batteries, providing insights into their internal conditions and degradation mechanisms. Although EIS cannot provide real-time estimations like Kalman filtering techniques, it offers the advantage of not requiring multiple cycles. It can typically be conducted in a few minutes at a single state of charge across a frequency range of 0.02 Hz–20 kHz. A three-electrode setup is often preferred for this process (Middlemiss et al., 2020).

Data collected through EIS is commonly analysed with advanced machine learning models to enhance life estimations. For instance, integrating EIS with GPR has achieved a maximum estimation deviation within 5.5% of actual values (Jiang et al., 2022). Additionally, combining methods such as a convolutional neural network (CNN), a bidirectional long short-term memory (BiLSTM), and improved Particle Swarm Optimisation (IPSO), also known as IPSO-CNN-BiLSTM, has performed up to 35% better than traditional GPR models (Li et al., 2022).

Other studies to improve SoH predictions have tried integrating EIS with advanced machine learning models, such as using a Fractional Order Equivalent Circuit Model (F-ECM) combined with the AutoGluon framework, achieving an RMSE of 2.12% and an MAE of 1.67% (Li et al., 2024). Ensuring adequate rest times and optimal current amplitudes is necessary for stable and reproducible EIS measurements in order to minimise transient effects and enhance signal-to-noise ratios (Azizighalehsari et al., 2023).

While testing methods for determining the SoH of batteries are varied, they have been limited by the need for a specialised human in the loop. However, research on batteries and robots has increased in popularity, with some robots able to perform tasks on batteries even better than humans (Hathaway et al., 2024). This, in consequence, has raised the possibility of applying methods previously limited to lab environments in industrial settings.

### 2.2 Robotics in battery testing and disassembly

Robotising tasks in disassembling EV batteries offers substantial economic and practical benefits. Recent research has shown that up to 57% of the pack-to-module (P2M) disassembly tasks for the Mitsubishi Outlander PHEV battery pack can be fully automated (Hathaway et al., 2024). This finding is based on a detailed technical analysis conducted by Hathaway et al., who carefully documented the manual disassembly process. Their study categorised tasks into three levels of autonomy—fully autonomous, semi-autonomous, and fully manual.

Among disassembly tasks, cutting and milling operations stand out as particularly critical in robotic disassembly, given their complexity and the variety of components they address. Recent advancements, such as those reported by Rastegarpanah et al. (2021b), have introduced innovative techniques like vision-based path planning using model predictive control (MPC). However, the diversity of material properties and the lack of standardisation across battery designs present ongoing challenges. To address this, recent research by (Hathaway et al., 2023) has employed adaptive learning-based approaches, enabling robots to adjust critical parameters—such as feed rate, depth of cut, and mechanical compliance—in real-time. This adaptability is essential for managing the safe and efficient robotic disassembly of diverse battery components, underscoring the importance of flexible cutting strategies.

Recent advancements have been made in automating diagnostic assessments and establishing standardised benchmarking procedures for battery recycling and testing platforms (Sommerville et al., 2021; Montes et al., 2022). Notable contributions include the development of the Strategic materials Weighting And Value Evaluation (SWAVE) matrix for quantifying material recovery value, which was done with a comprehensive analysis of 44 commercial recyclers and the categorisation of recycling processes into four primary functions (Sommerville et al., 2021). These methods provide a structured framework for evaluating resource recovery within the battery recycling industry.

Another significant advancement in robotic testing of EV batteries is detailed in Rastegarpanah et al. (2021a). The proposed testbed incorporated a Franka robot arm for interfacing the EOAT with the terminals of a Nissan Leaf battery module, while a second Franka arm provided visual sensory feedback to support the operations of the first robot. To ensure precise and stable contact with the battery terminals, the control system dynamically switched from visual servoing to impedance control. This approach combined Cartesian impedance control with joint-level torque sensing to achieve compliance and stability during terminal engagement. The robotic framework, designed for Electrochemical Impedance Spectroscopy (EIS) testing, demonstrated an 83% success rate across 30 trials. This proof-of-concept underscores the potential for scalable and automated battery testing solutions, offering high accuracy with minimal human intervention.

In a recent study, (Rastegarpanah et al., 2024b) demonstrated a novel approach to sorting disassembled EV battery components using their developed Decoupled Hybrid Visual Servoing (DHVS) method. This method combines the advantages of Image-Based Visual Servoing (IBVS) and Position-Based Visual Servoing (PBVS) while addressing their respective limitations. The study also incorporated the ITRA software interface to further showcase the sorting process for disassembled EV battery components, as illustrated in Figure 1. This integrated framework highlights the potential for efficient and precise sorting in automated disassembly systems.

Similarly, the study by Tan et al. (2021) presents a hybrid disassembly framework for EV batteries, addressing the inefficiencies, high costs, and safety hazards of current manual disassembly processes by incorporating automated robotic arms and specialized tools. This framework optimised design, safety, and cost parameters, allowing for a more efficient and safer disassembly process. Qu et al. (2024) have developed platforms that employed four industrial robots to automate the disassembly of plug-in hybrid EV battery packs, applying them to non-destructive tasks. Additionally, studies emphasise the importance of human-robot collaboration in EV battery disassembly, advocating for a flexible approach that combines manual and automated processes to optimise safety and productivity, especially in hazardous environments (Villagrossi and Dinon, 2023).

While robotic approaches for EV battery disassembly have seen significant development, the application of robotic systems for SoH diagnostics remains underexplored, particularly at an industrial scale. The SoH diagnostics are critical for determining which batteries are still viable for use and which should be recycled, making the disassembly process crucial for effectively managing the end-of-life stage of EV batteries. Many EIS tests are conducted at a lab scale, but with the growing number of retired EV batteries, there is a pressing need for scalable solutions for industrial-level testing. Hence, this implementation is a proof of concept demonstrating how automated testing using EIS can be fully established across the industry. The subsequent sections will detail the implementation, including the tools, procedures, control architecture, and the findings and results of the testing conducted using the industrial setup.

## 3 Methodology and experimental setup

Our experimental design focuses on implementing EIS within an automated robotic setup that guides the field for battery diagnostics. With this study, there are two purposes: first, the aim to improve the automation of battery testing for industrial settings through EIS technology. Second, to unveil the capabilities of EIS, which demonstrates great promise for a more precise, effective, and scalable SoH assessment of batteries.

### 3.1 Task description

Our proposed robotic framework for EIS testing involves five subtasks: **1.** Moving the robot to the task space, close to the battery terminals, using ITRA (Mineo et al., 2019; Rastegarpanah et al., 2024b). **2.** Switching from pre-programmed movements to an admittance control in close proximity to contact with the battery terminals to add compliance to the system and perform better precision movements accordingly. **3.** Collecting EIS data by a potentiostat connected to the EOAT. **4.** Safe disconnection from the battery terminals and returning to the home position. **5.** Analysis of the collected EIS data.

#### 3.1.1 Setup for EIS testing

**FIGURE 2:**
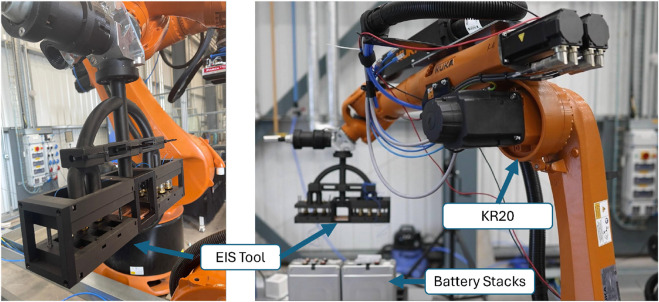
The KUKA KR20 robot is mounted on a 5 m rail and equipped with custom-designed tooling for connecting battery terminals to the potentiostat.

**FIGURE 3:**
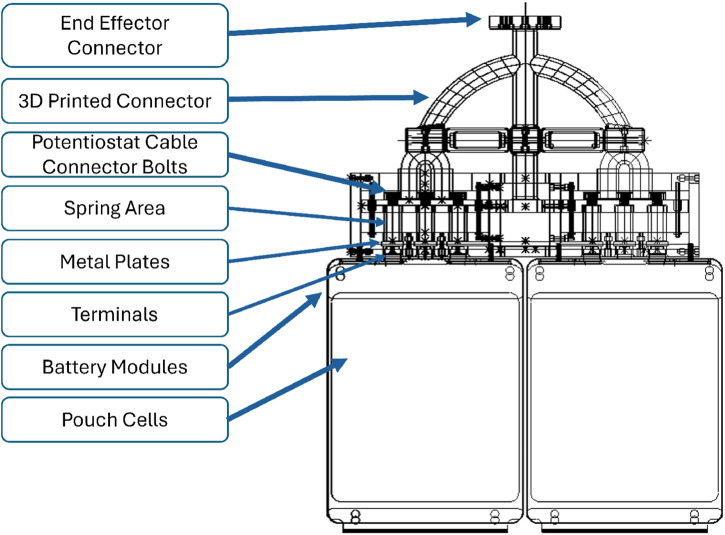
Detailed view of the 3D CAD model of the robot’s End-of-Arm tooling and the stack of LIB modules. Metal plates in the connection box are supported by springs to provide extra compliance in contact with the LIB terminals, allowing connection to two modules simultaneously.

The experimental setup consisted of a KUKA KR20 mounted on a 5 m rail, chosen for its range of movement due to the large workspace and load capacity to hold the designed EOAT Potentiostat (Figure 2). The EOAT, shown in Figure 3, has a main body 3D printed with PLA and metal plates made of aluminium 6061. The reliability and safety were validated by conducting finite element analysis on the proposed end-effector design.

The robot also had a force-torque sensor attached to the end-effector to enable the use of an admittance control. The integration of ITRA running within a remote computer allowed precise pre-programmed coordinates to be fed to the robotic system while also enabling the use of the admittance control.

A Nissan Leaf battery pack consisting of 24 modules is placed in the testing area next to the robot. Before initiating tests, the batteries underwent a preparation phase performed by a human technician, including cleaning terminals and performing voltage checks to ensure their correct functioning. An alarm system was designed and connected to the EOAT to ensure proper connection between the potentiostat and the battery modules by activating a series of LEDs on the EOAT and a piezo buzzer, which emits a loud sound upon successful connection. The VMP3 Multichannel Potentiostat and FlexP060 from BioLogic were used for EIS testing. Once the connection between the EOAT and the battery terminals is established, an operator runs the data capture software and starts the collection process. Once the testing is completed, the robot disconnects from the battery and returns to a defined home position.

**FIGURE 4:**
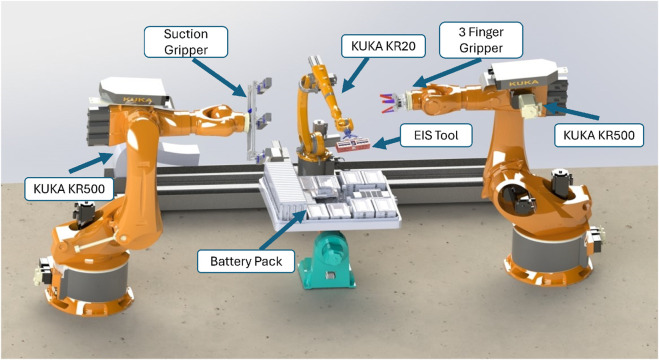
The digital twin designed to replicate the movements of the robots.

The robot control system was implemented using a computer featuring a GTX 1080 Ti graphics card, an Intel i7 processor, and 32 GB of RAM. The computer was linked to the controller of a KUKA KR20, running a KUKA Robot Language (KRL) module that contained all required KUKA configurations to enable the execution of the ITRA functions. Additionally, a digital twin environment was developed in SolidWorks to enable users to visualize the robot’s movements prior to executing commands in the physical environment. The SolidWorks simulation is illustrated in Figure 4.

#### 3.1.2 Safety protocols

Before the tests, a risk assessment was conducted for every component of the testing robotic cell. Instruction and safety protocols from our previous research (Rastegarpanah et al., 2021a) were followed and adapted to the industrial environment for the testing. The following points were considered: a) A thermal image camera was set up next to the cell to monitor the temperature of the battery modules during the experiment to mitigate the risks. b) The robotic cell is enclosed within a safety enclosure equipped with an interlock mechanism, ensuring operational safety. The system triggers an immediate shutdown of all robots when the enclosure door is opened, preventing unauthorized access during operation. c) Insulating gloves were worn to connect the cables of the Potentiostat to the EOAT. d) The Kuka KR20 robot was set in T1 mode, i.e., controlled in manual mode with speed limits, requiring an operator to continuously press the operation button for the robot to perform the tasks. e) Multiple robot emergency buttons were set up, including those in the teach pendant and external to it, to stop the robot in case of emergencies. f) Equipping the robotic cell with multiple buckets of sand to quickly submerge modules in case of fire, excessive temperature rise, smoke, or other thermal events. This is a crucial safety precaution when working with LIB. g) Mockup 3D-printed batteries were utilized to validate the procedure before testing with active batteries, allowing verification of the KUKA KR20’s movements.

#### 3.1.3 Robot control architecture

A strategy was developed to control the KR20 robot arm equipped with the EOAT and move it toward the battery terminals as a step toward full automation. Initially, the KR20 was controlled in joint space, making joint movements to predefined positions within the workspace to ensure both safety and precision. These positions included the following: i) the home position, where the robot is fully retracted and in a safe state; ii) the approach position, a midway point where the robot begins to decelerate as it nears the battery; iii) the battery reach position, a critical point 10 cm apart from the battery terminals where control is switched to manual mode, allowing the admittance controller to make the movement granular and ensure safe contact with the terminals; iv) the battery retreat position, where the robot withdraws from the terminals after completing the task, and finally, the return to the home position, where the robot is once again fully retracted and in a safe state. This sequence of positions (Home, Approach, Reach, Retreat, and Home) ensures controlled and safe operation, allowing for the integration of the admittance control during the critical battery reach phase.

The robot’s velocity varied depending on the position it aimed to reach. When moving from the home position to the pre-defined approach position or from the retreat position back to the home position, the robot operated at 50% of T1 mode speed. From the approach position to the reach position and from the reach position to the retreat position, the robot operated at 20% of T1 mode speed. While in admittance control mode, the robot was adjusted to allow a maximum of 5% of the maximum velocity of T1 mode. These percentages were chosen as part of the safety procedure.

ITRA makes communication to the computer and integration with the sensors possible. ITRA allows a high-level programming language (Python and Matlab, among others) to communicate with our robotic setup. The KUKA KR20 can be operated with ITRA with a control mode that relies on the KRL and the teach pendant a keyboard or predefined positions. ITRA establishes communication with MATLAB by running a script in the teach pendant and the connected computer. While operating in the KRL mode, target positions are selected and saved as waypoints, with the robot reaching them in the chosen order, using either Cartesian or joint space depending on the selected configuration; for the implementation, linear Cartesian movements were chosen as they are easier for a human operator to control, plan, and visualize compared to joint movements (Craig, 2005). Adjustments to the predefined positions were made over the experiments.

ITRA also provides a real-time control mechanism for contact with the battery terminals. This is called the RSI-based approach, exploiting the KUKA Robot Sensor Interface (RSI) (KUKA Robotics, 2014) and allowing commands to be sent back and forth at a 4 ms rate. The system continuously measures forces using a Force-Torque sensor mounted between the EOAT and the robot’s end-effector. These forces are captured in real-time and transmitted back to the central control computer via the RSI at a 4 ms interval, ensuring that the system has an up-to-date understanding of the interaction forces at all times. The control system then evaluates the incoming force data against predefined thresholds, which are set based on the safety requirements of the task and the mechanical limits of the robot and the environment, such as the fragility of battery terminals. The robot continues its pre-programmed motion path if the forces remain within acceptable limits. However, the admittance controller intervenes if the forces exceed the established thresholds, this meaning, that the robot will automatically go to a pre-programmed position to make contact with the battery terminals, but will adjust its position dependent on the generated forces.

**FIGURE 5:**
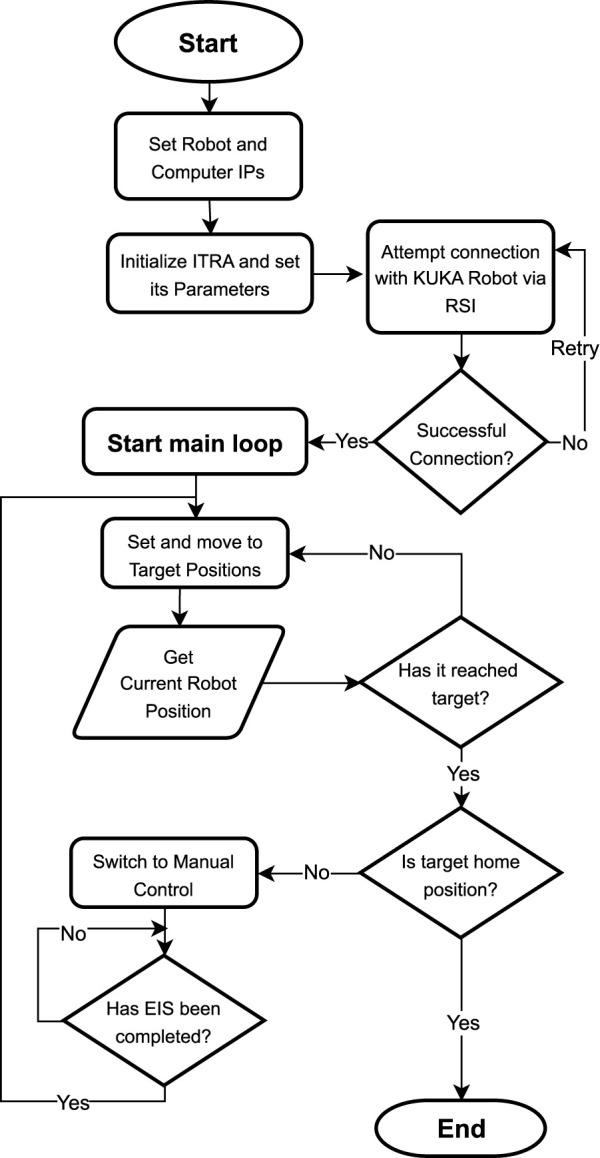
Flowchart of Robot Control Architecture for EIS data acquisition.

The controller dynamically adjusts the robot’s motions by modifying the trajectory, reducing the speed, or retracting the end-effector to prevent damage. These adjustments are computed in real-time and immediately communicated to the robot via RSI, allowing the robot to respond promptly to varying conditions during the battery terminal connection process. At the same time, the controller via the teach pendant or a keyboard allows the operator to move the end-effector, with the same trajectory adjustments relying on the admittance controller, being this approach preferred over the pre-programmed positions, due to positional errors that can arise. Figure 5 shows the control procedure performed by ITRA for positioning the KR20 in the industrial cell, EIS data acquisition, and movement back to the home position.

The admittance control strategy focuses on how the end-effector position and velocity of the robot are influenced by the external forces applied by the environment. The KR20 robotic arm has 6 degrees of freedom (DOF), so n=6 . Mathematically, this relationship is represented by Equation 1:

$$F\left(t\right)=m\ddot{x}\left(t\right)+b\dot{x}\left(t\right)+kx\left(t\right)$$ (1)

where $m\in R^{n\times n}$ is the virtual mass matrix, $b\in R^{n\times n}$ is the adaptive damping coefficient matrix, $k\in R^{n\times n}$ is the adaptive stiffness matrix, $x\left(t\right)\in R^{n}$ represents the position vector of the robot’s end-effector at time t , $\dot{x}\left(t\right)\in R^{n}$ is the velocity vector of the robot’s end-effector at time t , $\ddot{x}\left(t\right)\in R^{n}$ is the acceleration vector of the robot’s end-effector at time t , The external force applied by the environment at time t is denoted by $F\left(t\right)\in R^{n}$ .

To incorporate adaptive gain into this control system, the damping matrix b(t) and stiffness matrix k(t) are made time-dependent, adapting based on real-time feedback from the external forces. These adaptive gains are introduced to dynamically modify the damping and stiffness matrices to ensure the robot adjusts its responsiveness in real-time. Specifically, the matrices are modified as shown in Equation 2.

$$b\left(t\right)=\alpha \left(t\right)\cdot b_{0}\;\text{and}\;k\left(t\right)=\beta \left(t\right)\cdot k_{0}$$ (2)

Here, $b_{0}\in R^{n\times n}$ and $k_{0}\in R^{n\times n}$ are the nominal damping and stiffness matrices, respectively, representing the system’s baseline behaviour. The adaptive gain factors α(t) and β(t) are scalar functions of time that adjust the system’s responsiveness based on real-time force feedback or position error. This allows the system to dynamically adjust its behaviour, making it more or less responsive to external forces, especially as the robot approaches delicate tasks, such as connecting to battery terminals.

To fully incorporate the adaptive gain into the governing Equation 1, the expressions for the time-dependent damping and stiffness matrices are substituted.

$$F\left(t\right)=m\ddot{x}\left(t\right)+\alpha \left(t\right)b_{0}\dot{x}\left(t\right)+\beta \left(t\right)k_{0}x\left(t\right)$$ (3)

This updated equation is shown in Equation 3, where $\alpha \left(t\right)b_{0}$ represents the adaptive damping term. The factor α(t) , with the damping gain calculation shown in Equation 4, adjusts the nominal damping matrix $b_{0}$ in real-time based on force feedback. The function α(t) ensures that the damping gain decreases as the external force F(t) approaches the maximum allowable force $F_{\text{max}}$ , making the system more responsive to smaller forces.

On the other hand, $\beta \left(t\right)k_{0}$ represents the adaptive stiffness term, with the calculation shown in Equation 5. However, the contribution of the stiffness gain is minimal because the position deviation $x_{e}\left(t\right)=x_{d}\left(t\right)-x\left(t\right)$ is very small, approaching zero but not exactly zero. Therefore, while the stiffness gain is still present, its effect on the system is negligible compared to the damping gain, and it contributes very little to overall control, as given by Equations 4, 5.

$$\alpha \left(t\right)=\max\left(\min\left(\alpha_{\text{max}},\frac{F_{\text{max}}-|F\left(t\right)|}{F_{\text{max}}}\cdot \alpha_{0}\right),\alpha_{\text{min}}\right)$$ (4)

$$\beta \left(t\right)=\max\left(\min\left(\beta_{\text{max}},\frac{x_{\text{threshold}}-|x_{e}\left(t\right)|}{x_{\text{threshold}}}\cdot \beta_{0}\right),\beta_{\text{min}}\right)$$ (5)

Equation 3 can be rearranged to solve for the acceleration $\ddot{x}\left(t\right)$ as given by Equation 6:

$$\ddot{x}\left(t\right)=m^{-1}\left(F\left(t\right)-\alpha \left(t\right)b_{0}\dot{x}\left(t\right)-\beta \left(t\right)k_{0}x\left(t\right)\right)$$ (6)

The position and velocity of the robot are updated iteratively using numerical integration. The velocity is updated as given by Equations 7, 8:

$${\dot{x}}_{d}\left(t+\Delta t\right)={\dot{x}}_{d}\left(t\right)+\ddot{x}\left(t\right)\Delta t$$ (7)

$$x_{d}\left(t+\Delta t\right)=x_{d}\left(t\right)+{\dot{x}}_{d}\left(t\right)\Delta t$$ (8)

With Δt being the discrete time step given by the 250 Hz limit of the data transfer rate.

To ensure accurate control, position and velocity errors are computed as given by Equations 9, 10:

$$x_{e}\left(t\right)=x_{d}\left(t\right)-x\left(t\right)$$ (9)

$${\dot{x}}_{e}\left(t\right)={\dot{x}}_{d}\left(t\right)-\dot{x}\left(t\right)$$ (10)

where $x_{d}\left(t\right)\in R^{n}$ is the desired position vector at time t , ${\dot{x}}_{d}\left(t\right)\in R^{n}$ is the desired velocity vector at time t , $x_{e}\left(t\right)\in R^{n}$ is the position error vector at time t , and ${\dot{x}}_{e}\left(t\right)\in R^{n}$ is the velocity error vector at time t .

These errors are used in a feedback loop to adjust the control inputs and improve the performance of the admittance controller. The parameters for the damping (b) and stiffness (k) were tuned through experimental trials. The tuning process involved adjusting these parameters iteratively to balance responsiveness and stability, ensuring that the robot could effectively respond to external forces without causing excessive oscillations or instability. A force-torque sensor is attached to the end-effector of the KR20, providing real-time force feedback. This feedback loop lets the operator monitor the forces applied to the battery terminals.

The operator uses a keyboard as an interface tool to translate manual inputs into end-effector movements. These movements are influenced by external forces, including the contact force from the battery terminals and gravity-generated forces, which must be accounted for during operation. The admittance controller uses real-time force inputs to adjust the position of the end-effector in response to the external forces, ensuring the system is not damaged during contact.

#### 3.1.4 Task procedure

To complete the task, everything is precisely coordinated to maintain high efficiency and safety. The robot only operates when the work cell’s doors are closed and the interlock system is activated, ensuring the safe operation of the KR20 system.

First, the robot is moved to its home position in T1 mode, ensuring it starts from the correct state. After verifying the robot’s position, the KR20 runs a script on its teach pendant, enabling communication with the RSI manager in the ITRA system for computer-based control and stopping.

The robot is then guided using the ITRA KRL Control Method, which employs a MATLAB script to move it to pre-defined Cartesian coordinates, which correspond to specific locations within the work cell. Throughout this process, the operator ensures safety by holding the teach pendant in T1 mode, with one hand positioned near the emergency stop button on the pendant, ready to intervene if necessary.

The system then safely approaches the coordinates with linear movements, carefully getting closer to the battery modules previously prepared in the work cell for easy connection. The admittance control loop activates when the KR20 reaches its final programmed coordinate, located near the battery terminals within the work cell. This control loop allows the system to respond dynamically to external forces measured by the force-torque sensor (as mentioned in Section 3.1.3), ensuring precise contact with the battery terminals.

The adaptive gain used in the control system has been explained in Equation 3, where the damping and stiffness matrices adjust based on real-time feedback. The control strategy ensures that the robot remains responsive to changing forces, preventing potential damage to both the robot and the battery modules. Specifically, if the forces exceed the predefined safe thresholds, set with a safety factor of 1.5 of the max forces obtained during testing a successful connection. The safety mechanism operates by dynamically adjusting the damping and stiffness matrices or, in more critical cases, immediately stopping the movement of the robot through emergency stop procedures integrated into the control loop.

At the 165-second mark of the test, the operator gains the ability to manually control the system using a keyboard or the teach pendant. The force-torque sensor provides continuous force feedback, enabling both the operator and the system to monitor and respond to any forces that deviate from expected parameters. Additionally, LEDs illuminate and buzzers sound when successful alignment and connection are achieved, providing further confirmation and acting as a safety net to prevent any harm to the battery terminals due to misalignment or excessive force. After a successful connection, the EIS data collection starts and continues for 25 min. Then, the robot separates from the battery modules and returns to its home position. The EIS testing description is detailed below.

#### 3.1.5 EIS testing procedure and data analysis

**FIGURE 6:**
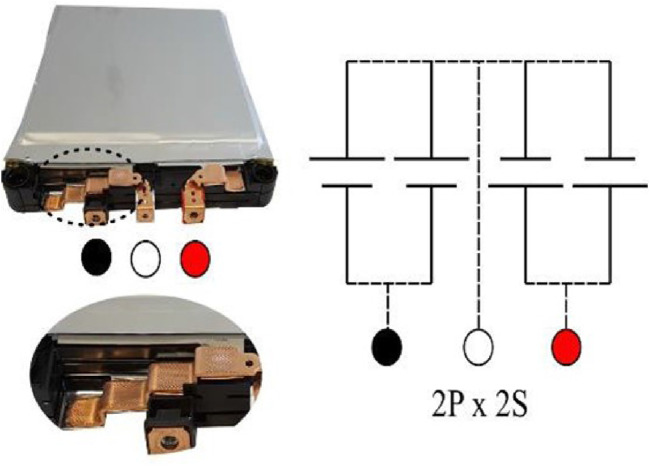
Battery Module Terminals diagram.

This test evaluates the state health of two Nissan Leaf battery modules connected in series using VMP3 Multichannel Potentiostat and FlexP060 from BioLogic. The pouch cells employed in this study utilise lithium-manganese-oxide with nickel oxide (LiMn2O4 with LiNiO2) as the cathode material. Each module has three terminals: red, white, and black (RWB). The red and black terminals function as the positive and negative terminals of the module, respectively. The white terminal is either a positive or negative terminal relative to the two arrangements, as illustrated in Figure 6. The cells’ terminals are welded to copper bus bars to establish electrical connections for the module, which prevents direct access to individual cells. Consequently, following pack disassembly, the smallest accessible unit in the module available for testing is the 2P pairs, either the RW or WB cell pairs, referred to as a cell pair. Each cell pair has an A hour rating of 66 Ah and a nominal voltage of 3.75 V. The module canister is characterised by dimensions of (303 × 223 × 35 mm) and a weight of 3.8 kg.

**FIGURE 7:**
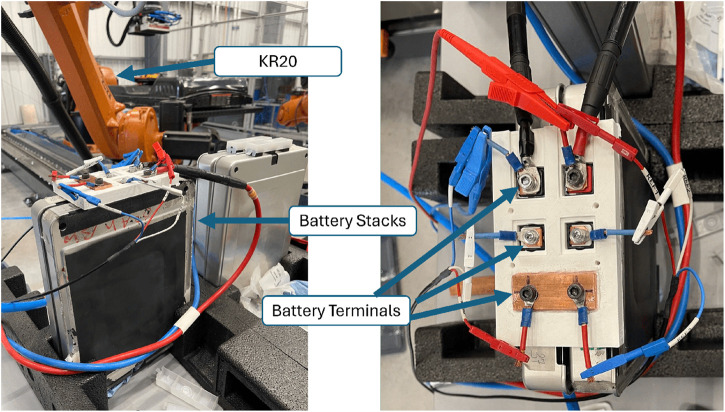
Connections between battery modules and the Potentiostat, prior to connection with the EOAT.

The modules were connected in series, as illustrated in Figure 7. This configuration was chosen to include modules with varying health conditions, offering a detailed assessment of the EIS test. It is worth noting that the adopted experimental design capitalises on the equipment’s capacity to assess modules in series, presenting an efficient and cost-effective alternative to individual testing.

The EIS test was initiated to assess their electrochemical performance after confirming a successful connection between the EOAT and the battery modules. The experiment aimed to gain information and data about the underlying electrochemical processes using a frequency range tailored for impedance measurements (1 kHz–20 mHz) in galvanostatic mode with 10 A perturbation current. Impedance spectra were recorded during the test, providing essential data for subsequent analysis to determine the SoH of each cell pair within the series connection of modules.

#### 3.1.6 Finite-element analysis

A Von Mises stress analysis was carried out, studying the effect the forces applied on the end-effector tool had when connecting to the terminal of the LIBs. The SolidWorks simulation modelled the Terminals and battery casing utilising Aluminum 6061 alloy. Majority of the body of the tool and the connectors interfacing with the robot were constructed from polylactic acid (PLA), while the bolts, nuts, and screws used in the tool were made from a steel alloy. Fixtures were added to the body of all LIBs, simulating them being fixed in position. Spring connectors were added to the springs within the EOAT, and a force of 15 N was applied to the end-effector (Figure 11). The Aluminium 6061 Alloy was defined as a Linear Elastic Isotropic Material with a Poisson’s ratio of 0.33 N/A, a density of 2.7 $g/cm^{3}$ and a tensile strength of 124.084 MPa. For the steel alloy, the properties were a Poisson’s ratio of 0.28 N/A, a density of 7.7 $g/cm^{3}$ , and a tensile strength of 723.826 MPa. A Poisson’s ratio of 0.35 N/A, a density of 1.3 $g/cm^{3}$ , and a tensile strength of 50 MPa were defined for the PLA.

## 4 Results and discussion

This section presents and analyses the experimental outcomes of our procedure, validating the EIS tests, mechanical integrity of the EOAT, and the robotic process carried out in the semi-autonomous test. Additionally, it highlights opportunities for future iterations of this work. Table 1 illustrates the measured capacity of each cell pair within the series connection.

**TABLE 1:** Cell Pair Capacities and State of Health. SoH is determined by comparing the current capacity with the initial rated capacity in Ah.

Cell pair	Capacity Ah/SoH%
Pair 1	43.27/65%
Pair 2	55/83%
Pair 3	60/90%
Pair 4	61/92%

### 4.1 Validation of the EIS experimentation

**FIGURE 8:**
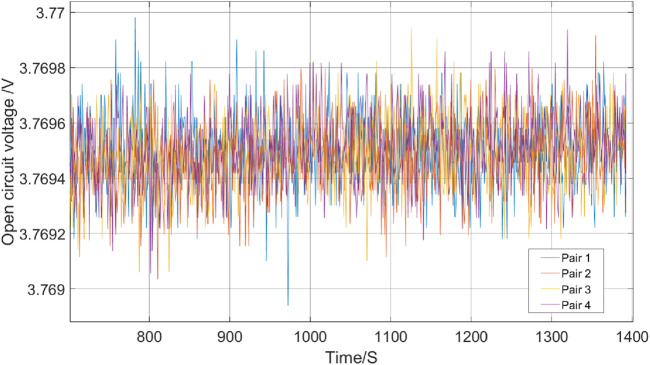
Voltage readings across different battery modules obtained using EIS. The plot shows the voltage stability during the test, which indicates a secure connection and consistent performance of the battery modules.

**FIGURE 9:**
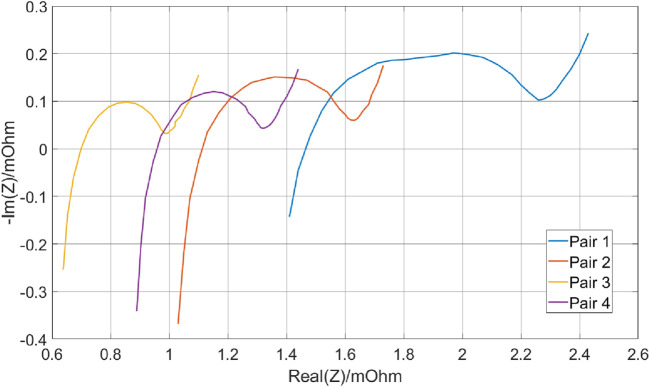
Internal resistance readings across different battery modules obtained using EIS. The plot highlights variations in internal resistance among the cell pairs, with Cell Pair 1 exhibiting the highest internal resistance. Conversely, Cell Pairs 3 and 4 show the lowest internal resistance.

As illustrated in Figure 8, the results demonstrate voltage readings consistent with expectations, signifying a secure and well-established connection. Figure 9 highlights that cell pair 1 exhibited the highest internal resistance, around 1.5 mΩ , indicative of the lowest capacity compared to other cell pairs within the same battery module. Conversely, cell pairs 3 and 4 exhibited the lowest internal resistance with resistances of 0.69 mΩ and 0.94 mΩ respectively, aligning with their anticipated high capacity. This consistency shows the effectiveness of EIS in identifying the health of individual cell pairs within multi-cell modules, thereby facilitating improved diagnostics and informed maintenance strategies for EV battery systems. The distinct characteristics observed in cell pair 1, such as its higher internal resistance than the other pairs, strongly indicate it as the weakest link in the battery module. This identification presents an advantage for battery re-use. It allows targeted interventions, such as replacement or specific maintenance procedures, to address the weaker cell pair and maintain overall battery performance.

Moreover, while EIS serves as a practical initial test for identifying individual cell pair health, it can be complemented by additional testing techniques for a more comprehensive assessment. One such technique is a capacity test, which provides insights into the remaining useful life and degradation mode of the aged cell pair. By conducting capacity tests on the identified weaker cell pair, engineers can better understand its current state and anticipate its future performance. This allows for proactive measures, such as adjusting charging protocols or scheduling timely replacements, to mitigate potential risks associated with degradation and ensure the longevity of the battery system.

### 4.2 Robotic performance

The semi-autonomous process presented no major complications, with pre-programmed positions easy to follow. The connection with the keyboard and ITRA real-time control allowed for the necessary manoeuvrability in close range. A schematic diagram of the robotic control process during different steps was illustrated in Figure 5. This diagram outlines the workflow from initial positioning using pre-programmed coordinates, transitioning to force control during the connection phase, and completing the EIS data collection. The EOAT, mounted on the KR20, was successfully used for the experimental procedure without major challenges thanks to its design (Figure 3) and the simulations carried out beforehand (Figure 4). Although the EIS data collection took 25 min, the ability of the robot to maintain stability during the connection was crucial for it, which the robot was able to perform with our setup.

**FIGURE 10:**
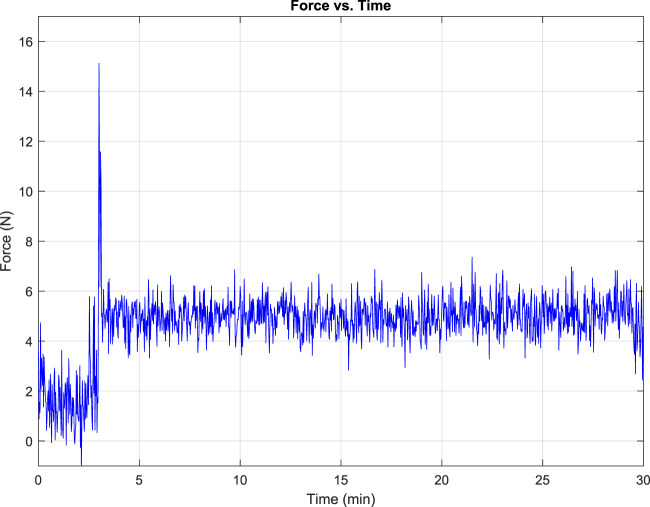
Resultant force present on EOAT at the KUKA KR20 when interacting with the LIBs terminals during the operation of the system.

Despite a significant force spike of 15.1 N during the operator’s connection to the batteries (Figure 10), this force was distributed among the battery terminals, reducing the risk of damaging the battery terminals. The force was recorded during the trials with the mock-up cells, and the data was then used to perform a finite-element analysis of the tool to carry out the procedure in the real battery cells.

### 4.3 Finite-element analysis for a damage-free contact

**FIGURE 11:**
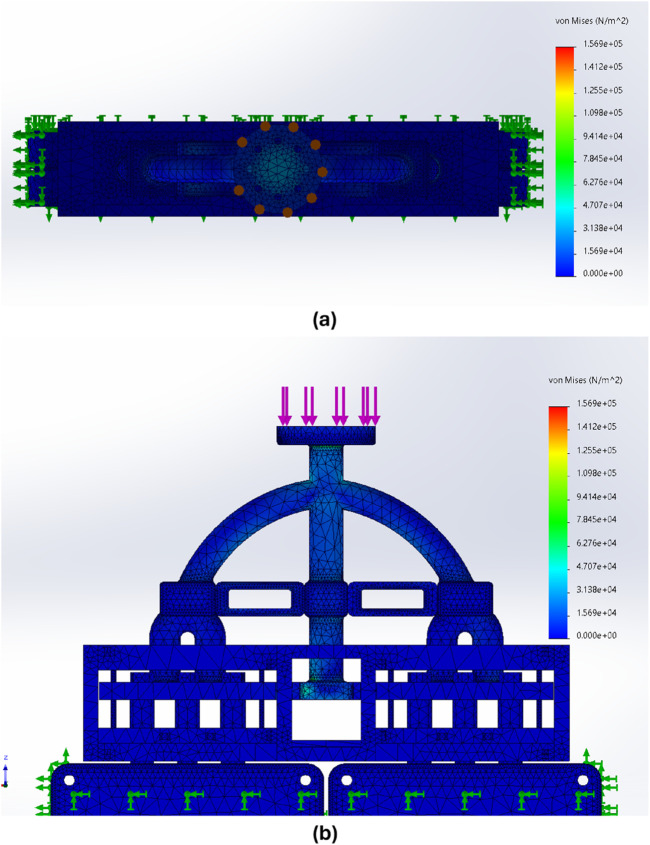
Results of Von Mises stress **(A)** Top view, depicting maximum forces in the connection **(B)** and front view depicting the forces in the centre of the tool.

The performed Finite-element analysis confirmed the tool’s usability and limits for the real-world experiment. A force of 15.1 N was applied in the FEA analysis since this was the maximum force presented while connecting with the LIBs terminals (Figure 11B.). Figure 11 shows the results of the Von Mises analysis in both Top View and Front View, with the maximum stress spots represented in their meshes with a maximum stress of 1.56905e + 05 $N/m^{2}$ . While the stress reached with the tool during contact with the batteries was insufficient to cause deformation, FEA indicated that the weakest part of the tool is a corner of the centre rod directly at the bottom, suggesting future iterations could benefit from structural improvements to distribute the forces better.

### 4.4 Scalability and industrial application

It has been demonstrated that the developed robotic framework can identify faulty modules, even when scaled up to multiple modules. This capability suggests that the framework can serve as a complementary step before full robotic disassembly, allowing for the identification of reusable modules and replacing those that should be discarded. This pre-disassembly testing ensures that only defective modules are targeted for replacement, optimizing the reuse and recycling process and enhancing its speed.

Our developed semi-automated process using industrial robots has proven effective for battery testing, offering significantly improved safety, precision, and efficiency over traditional methods. A possible enhancement would be integrating more advanced control or haptic systems. These approaches could improve the system’s responsiveness and adaptability. Advanced control systems might enhance the robot’s ability to handle variations in module positions or unexpected forces, making the process more robust. On the other hand, haptic systems would allow the robot to “feel” the forces during connection and disconnection, providing real-time feedback for even more precise manipulation.

Additionally, machine vision algorithms could improve the system by enabling better object recognition and localisation. For example, machine vision could detect and map the module positions accurately, as demonstrated by (Aflakian et al., 2023), who applied deep reinforcement learning (RL) with human demonstrations to optimize robotic tasks. The authors constrain the action space for a mixture agents by applying 3D convex hulls, thereby reducing the search space and enhancing learning speed and resilience to local minima. Incorporating machine vision with reinforcement learning could expedite our robot’s ability to identify and handle battery modules in varying conditions. Finally, connecting the robotic system to our VR platform (Rastegarpanah et al., 2024a) would allow for real-time monitoring and control, enabling operators to train and adjust the system’s behaviour in a virtual environment before real-world deployment.

## 5 Conclusion

In this study, we extended our previous work, where vision-guided manipulation techniques were used to predict the SoH of a single battery module, by demonstrating how industrial robots can predict the SoH for a stack of modules simultaneously. This innovation highlights the potential of robotic automation to accelerate the battery testing process, reduce testing time, and advance sustainability by enabling battery reuse and promoting the circular economy.

Our semiautomatic robotic system, leveraging EIS, demonstrated precision in identifying battery degradation, with the most degraded cells reaching an internal resistance of 1.5 mΩ . The robot successfully distinguished less degraded batteries while maintaining safe operation with maximum contact forces no larger than 15.1 N. The system’s pipeline of subtasks ensures safety and scalability, offering a streamlined and replicable process for accessing and testing battery packs, modules, and cells, which can be automated in future applications.

By integrating automation, this system not only minimizes labor costs and improves efficiency but also supports environmental sustainability by reducing LIB waste and extending battery life. These advancements facilitate early SoH detection and enable second-life applications like energy storage, reducing the environmental footprint of raw material extraction and overproduction.

In future, we aim to fully automate the process by incorporating visual servoing and advanced control systems, enabling autonomous connection to battery terminals and initiating automated data collection. This progress will further enhance the system’s industrial applicability and extend its benefits to a broader range of battery types, cementing the role of robotic automation in sustainable battery lifecycle management.

## Data Availability

The raw data supporting the conclusions of this article will be made available by the authors, without undue reservation.
